# Association between the triglyceride-glucose index and its combined obesity indicators and the risk of hypertension in middle-aged and older Chinese adults: A nationwide cross-sectional study

**DOI:** 10.1371/journal.pone.0316581

**Published:** 2025-01-02

**Authors:** Huoping Zheng, Meiling Xu, Jing Yang, Mingjun Xu

**Affiliations:** Department of Endocrinology and Metabolism, The Third People’s Hospital of Jingdezhen, Jingdezhen, China; Tehran University of Medical Sciences, ISLAMIC REPUBLIC OF IRAN

## Abstract

**Objective:**

This study aimed to explore the association between the triglyceride-Glucose Index (TyG) and its combination with obesity indicators in relation to the risk of hypertension among middle-aged and elderly individuals in China.

**Methods:**

A total of 9,248 participants aged 45 and above were selected from the 2015 China Health and Retirement Longitudinal Study. Data on basic demographics, behavioral habits, medical history, physical examination results, and blood indicators were collected. The TyG and its obesity-related indicators, including TyG-BMI, TyG-WC, and TyG-WHtR were analyzed. These indicators were categorized into four groups based on quartiles, and the prevalence of hypertension within each group was assessed. Logistic regression analysis was conducted to evaluate the association between different TyG indices and obesity-related indicators with the risk of hypertension. Additionally, a restricted cubic spline model was employed to investigate the dose-response relationship between the TyG index, its combined obesity indicators, and the risk of hypertension. The receiver operating characteristic curve was utilized to determine the optimal predictive index for diagnosis.

**Results:**

This study included a total of 9,248 middle-aged and elderly individuals aged 45 and above, comprising 4,274 males (46.21%), with an average age of 61.38 ± 9.28 years. The prevalence of hypertension exhibited an increasing trend as the quartiles of TyG, TyG-BMI, TyG-WC, and TyG-WHtR rose. After fully adjusting for covariates, comparisons between the first quartile (Q1) and the fourth quartile (Q4) of TyG, TyG-BMI, TyG-WC, and TyG-WHtR revealed that all these metrics at Q4 level were associated with an increased prevalence of hypertension. The odds ratios were as follows: TyG: OR = 1.80 (95% CI: 1.48–2.19); TyG-BMI: OR = 5.09 (95% CI: 4.33–5.98); TyG-WC: OR = 3.96 (95% CI: 3.38–4.65); TyG-WHtR: OR = 3.91 (95% CI: 3.33–4.60). A linear correlation was observed between TyG and the risk of hypertension (P for non-linearity = 0.2267), while non-linear correlations were noted between TyG-BMI, TyG-WC, and TyG-WHtR with the risk of hypertension (P for non-linearity < 0.001). The receiver operating characteristic curve indicated that TyG-WC demonstrated the highest diagnostic performance for hypertension, with an area under the curve of 0.642 (95% CI: 0.631–0.654).

**Conclusion:**

As the levels of TyG, TyG-BMI, TyG-WC, and TyG-WHtR increased, the risk of hypertension among middle-aged and elderly individuals aged 45 and above in my country rises significantly. Among them, TyG-WC can be a predictor of hypertension in middle-aged and elderly people.

## Introduction

With China’s economic and social development, alongside the continuous improvement of health service levels, the average life expectancy of residents has steadily increased. Consequently, the survival period of patients with chronic diseases has also been extended. Moreover, factors such as population aging, urbanization, accelerated industrialization, and the prevalence of behavioral risk factors have significantly impacted the incidence of chronic diseases. As a result, the number of patients with chronic diseases in our country is expected to continue to rise, and the challenges associated with chronic disease prevention and control remain severe [1]. Hypertension, one of the most prevalent chronic diseases in our nation, has seen its prevalence increase annually [2]. Specifically, the prevalence of hypertension in the Chinese population has escalated from 5.1% in 1958–1959 to 23.2% in 2012–2015, leading to a substantial economic burden of disease [3]. As of 2019, there are 270 million patients with hypertension in our country, with more than 2 million individuals succumbing to complications related to elevated blood pressure each year, resulting in direct medical costs amounting to 36.6 billion yuan [4]. Therefore, identifying factors that predict the risk of hypertension is crucial for promoting early prevention of the disease.

Insulin resistance is a condition characterized by a diminished sensitivity and response to insulin, leading to an impaired ability of insulin to facilitate glucose transport into cells. This impairment results in metabolic abnormalities, such as hyperglycemia [5]. Insulin resistance is significantly implicated in the development and progression of hypertension and is regarded as a predictive factor for this condition [6,7]. The hyperinsulinemia-euglycemic clamp technique is recognized as the gold standard for assessing insulin resistance. However, its implementation in clinical practice is often hindered by technical complexity, high costs, and ethical considerations [8]. Previous research has indicated that the triglyceride-glucose index (TyG), derived from triglyceride (TG) and fasting plasma glucose (FPG) levels, serves as a valuable indicator for evaluating insulin resistance. In contrast to traditional markers of insulin resistance, the TyG calculation method is straightforward, cost-effective, and easy to measure [9]. Studies have demonstrated that TyG can effectively predict the incidence of hypertension within the Chinese population [10].

Obesity has been recognized by the World Health Organization as one of the top ten chronic diseases, significantly increasing the risk of various other chronic conditions, such as dyslipidemia, hypertension, diabetes, coronary heart disease, and myocardial infarction [11]. A cross-sectional real-world study involving 15.8 million adults revealed that the prevalence of obesity among adults in my country was 14.1% in 2019, representing a serious public health issue [12]. Obesity is closely associated with the development of insulin resistance and hypertension [13,14]. Previous research has indicated that the triglyceride-glucose (TyG) index, when combined with obesity indicators, is more effective than the TyG index alone in assessing insulin resistance and cardiovascular risk [15]. However, there is a paucity of studies examining the relationship between the combined TyG index and obesity indicators and the risk of hypertension among middle-aged and elderly individuals in my country. Therefore, this study aims to utilize the 2015 China Health and Retirement Longitudinal Study (CHARLS) as the data source to investigate the association between the TyG index, its combination with obesity indicators, and the risk of hypertension, thereby providing a scientific basis for the early prevention of hypertension in the elderly population of my country.

## Methods

### Participants

The data for this study were obtained from the CHARLS conducted in 2015 [16–18]. The survey aims to collect a representative set of high-quality micro-data encompassing middle-aged and elderly families and individuals aged 45 and above in China, with the goal of analyzing the country’s population aging problem, promoting interdisciplinary research on aging issues, and providing a more scientific basis for formulating and improving relevant policies. In 2015, CHARLS surveyed a total of 21,096 individuals. Exclusion criteria included: the absence of collected blood indicators, blood indicators not collected on an empty stomach, missing data on the TyG index and obesity-related indicators, and missing data on outcome indicators or covariates. Ultimately, 9,248 individuals were included in the final analysis. The CHARLS project received approval from the Peking University Biomedical Ethics Committee (IRB00001052-11015), and all research subjects provided informed consent.

### Research content

The study primarily involves a questionnaire survey, physical examination, and the collection of blood indicators. The questionnaire encompasses various demographic and health-related variables, including sex, age, residence, education level, marital status, smoking status, drinking status, and self-reported medical history, specifically regarding hypertension, diabetes, and dyslipidemia. The physical examination includes measurements of height, weight, waist circumference (WC), systolic blood pressure, and diastolic blood pressure. Blood indicators assessed in the study consist of triglycerides (TG), fasting plasma glucose (FPG), total cholesterol (TC), low-density lipoprotein cholesterol (LDL-C), high-density lipoprotein cholesterol (HDL-C), glycosylated hemoglobin (HbA1c), and uric acid (UA).

### Related indicator calculation formulas

Obesity levels were assessed using Body Mass Index (BMI), WC, and Waist Height Ratio (WHtR). Insulin levels were evaluated through the TyG index. The TyG index and obesity markers were combined to form TyG-BMI, TyG-WC, and TyG-WHtR. The calculation formulas for the relevant indicators are as follows: (1) WHtR = WC (cm) / height (cm); (2) BMI = weight (kg) / height^2^ (m^2^); (3) TyG = ln[TG (mg/dl) × FPG (mg/dl) / 2]; (4) TyG-BMI = TyG × BMI; (5) TyG-WC = TyG × WC; (6) TyG-WHtR = TyG × WHtR.

### Indicator definition

The diagnostic criteria for hypertension include a systolic blood pressure of ≥140 mmHg and/or a diastolic blood pressure of ≥90 mmHg, assessed without the use of antihypertensive medications, or through self-reported hypertension [19]. Dyslipidemia is diagnosed when TC is ≥240 mg/dL, TG is ≥150 mg/dL, LDL-C is ≥160 mg/dL, and HDL-C is ≤40 mg/dL, also without the use of blood lipid-lowering medications. The presence of one or more of these four indicators, along with abnormal or self-reported dyslipidemia, confirms the diagnosis [20]. Type 2 diabetes is defined as a fasting blood glucose level of ≥126 mg/dL or self-reported diabetes, again without the use of antidiabetic medications [21].

### Statistical analyses

Data analysis was conducted using SPSS 14.0 and R 4.3.2 statistical software. Measurement data conforming to a normal distribution were expressed as $\bar{x}$ ± s, and a t-test was employed for group comparisons. For measurement data not conforming to a normal distribution, results were presented as P_50_ (P_25_, P_75_), with a non-parametric rank-sum test utilized for group comparisons. Categorical data were expressed as constituent ratios or rates, and the χ^2^ test was applied for group comparisons. Additionally, a logistic regression model was employed to analyze the associations between different TyG indices, obesity combination indices, and the risk of hypertension. To elucidate the dose-response relationship between various TyG indices and their corresponding obesity combination indices with respect to hypertension risk, restricted cubic spline models were constructed with three nodes at P_10,_ P_50_, and P_90_. The diagnostic values were evaluated using Receiver Operating Characteristic (ROC) curves, and the predictive ability of different TyG indices and obesity combination indices for hypertension was quantified using the Area Under Curve (AUC). A two-sided test was performed with a significance level of α = 0.05.

## Result

### TyG index and its basic characteristics of participants combined with obesity indicators

**Fig 1:**
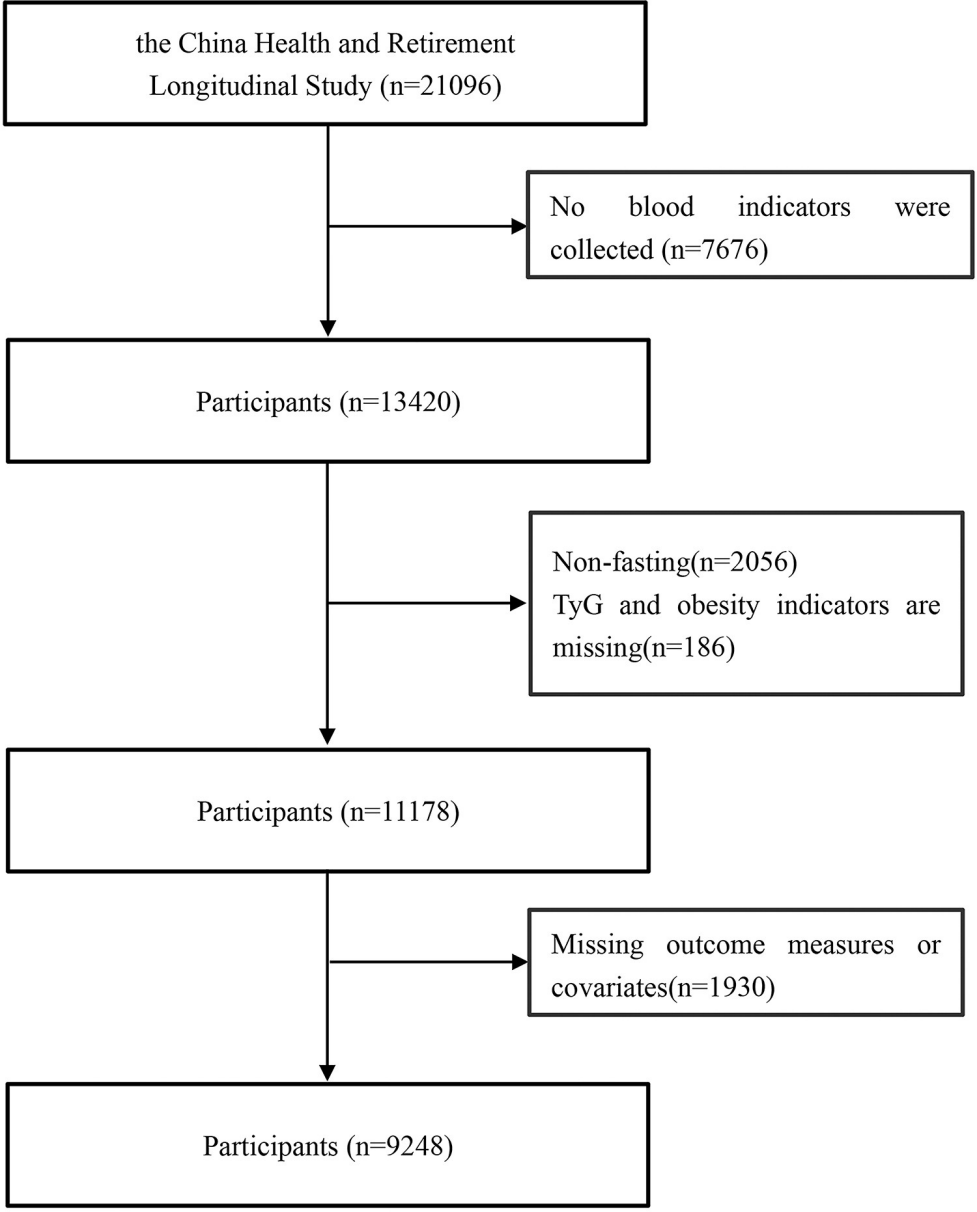
Process of inclusion of research subjects.

In 2015, CHARLS surveyed a total of 21,096 individuals. After excluding participants due to the absence of fasting blood indicators, as well as those with missing related data, a final sample of 9,248 individuals was included for analysis. Among these participants, the average age was 61.38 ± 9.28 years, and 4,274 (46.21%) were male. Significant differences were observed across different quartiles of the TyG, TyG-BMI, TyG-WC, and TyG-WHtR metrics in relation to gender, residential location, education level, marital status, smoking habits, alcohol consumption, and medical history, including hypertension, dyslipidemia, and diabetes. Additionally, there were statistical differences in age, BMI, waist circumference, WHtR, HDL-C, LDL-C, TC, FPG, UA, HbA1c, and TG (P < 0.05). For further details, please refer to Table 1, Fig 1, and Tables S1-S3 in S1 Appendix.

**Table 1:** Basic characteristics of different TyG quartiles.

Variable	Overall	TyG quartiles				P
		Q1	Q2	Q3	Q4	
Sex						<0.001
Male	4,274 (46.21)	1,303 (56.65)	1,054 (45.89)	984 (42.00)	933 (40.42)	
Female	4,974 (53.79)	997 (43.35)	1,243 (54.11)	1,359 (58.00)	1,375 (59.58)	
residence						<0.001
City	1,886 (20.39)	347 (15.08)	421 (18.33)	530 (22.62)	588 (25.48)	
Rural	7,362 (79.61)	1,953 (84.92)	1,876 (81.67)	1,813 (77.38)	1,720 (74.52)	
Education						0.013
Illiteracy	2,382 (25.75)	588 (25.57)	621 (27.04)	601 (25.65)	572 (24.78)	
Primary school	3,904 (42.21)	1,024 (44.52)	971 (42.27)	946 (40.38)	963 (41.73)	
Middle school and above	2,962 (32.03)	688 (29.91)	705 (30.69)	796 (33.97)	773 (33.49)	
Marital status						<0.001
Married	7,969 (86.17)	1,982 (86.17)	1,917 (83.46)	2,046 (87.32)	2,024 (87.69)	
Others	1,279 (13.82)	318 (13.83)	380 (16.54)	297 (12.68)	284 (12.31)	
Smoking status						<0.001
No	5,503 (59.50)	1,199 (52.13)	1,351 (55.82)	1,480 (63.17)	1,473 (63.82)	
Yes	2,736 (29.58)	841 (36.57)	723 (31.48)	595 (25.39)	577 (25.00)	
Quit smoking	1,009 (10.92)	260 (11.30)	223 (9.70)	268 (11.44)	258 (11.18)	
Drinking status						<0.001
No	6,039 (65.30)	928 (40.35)	790 (34.39)	755 (32.22)	736 (31.89)	
Yes	3,209 (34.70)	1,372 (59.65)	1,507 (65.61)	1,588 (67.78)	1,572 (68.11)	
Hypertension						<0.001
No	5,113 (55.29)	1,508 (65.57)	1,375 (59.86)	1,230 (52.50)	1,000 (43.33)	
Yes	4,135 (44.71)	792 (34.43)	922 (40.14)	1,113 (47.50)	1,308 (56.67)	
Dyslipidemia						<0.001
No	5,549 (60.00)	2,097 (91.17)	1,940 (84.45)	1,360 (58.05)	152 (6.59)	
Yes	3,699 (40.00)	203 (8.83)	357 (15.55)	983 (41.95)	2,156 (93.41)	
Diabetes						<0.001
No	8,008 (86.59)	2,219 (96.48)	2,144 (93.33)	2,053 (87.62)	1,592 (68.98)	
Yes	1,240 (13.41)	81 (3.52)	153 (6.67)	290 (12.38)	716 (31.02)	
Age	61.38±9.28	61.56±9.63	61.75±9.52	61.53±9.16	60.67±8.76	<0.001
BMI	23.83±3.54	22.11±3.15	23.28±3.35	24.33±3.38	25.56±3.32	<0.001
WC	85.76±11.11	80.98±9.49	84.00±10.55	87.16±11.40	90.85±10.41	<0.001
WHtR	0.54±0.07	0.51±0.06	0.53±0.69	0.55±0.07	0.58±0.07	<0.001
HDL-C	51.44±11.53	56.59±12.46	53.19±11.32	50.22±10.08	45.82±9.23	<0.001
LDL-C	102.94±28.94	94.72±25.79	105.00±27.43	109.48±28.53	102.42±31.67	<0.001
TC	184.52±36.50	169.01±31.40	180.83±32.87	188.99±33.97	199.11±40.31	<0.001
FPG	100.56±30.11	87.54±10.88	93.67±11.81	99.57±17.80	121.39±49.04	<0.001
UA	4.92±1.41	4.60±1.30	4.76±1.37	5.00±1.39	5.32±1.46	<0.001
HbA1c	5.99±1.00	5.70±0.47	5.78±0.51	5.94±0.76	6.54±1.60	<0.001
TG	111.50 [81.42,163.72]	67.26 [58.41,76.11]	95.57 [86.73,105.31]	134.51 [118.58,152.21]	222.12 [181.42,290.26]	<0.001

### Prevalence of hypertension by TyG index and its combination with obesity indicators

**Fig 2:**
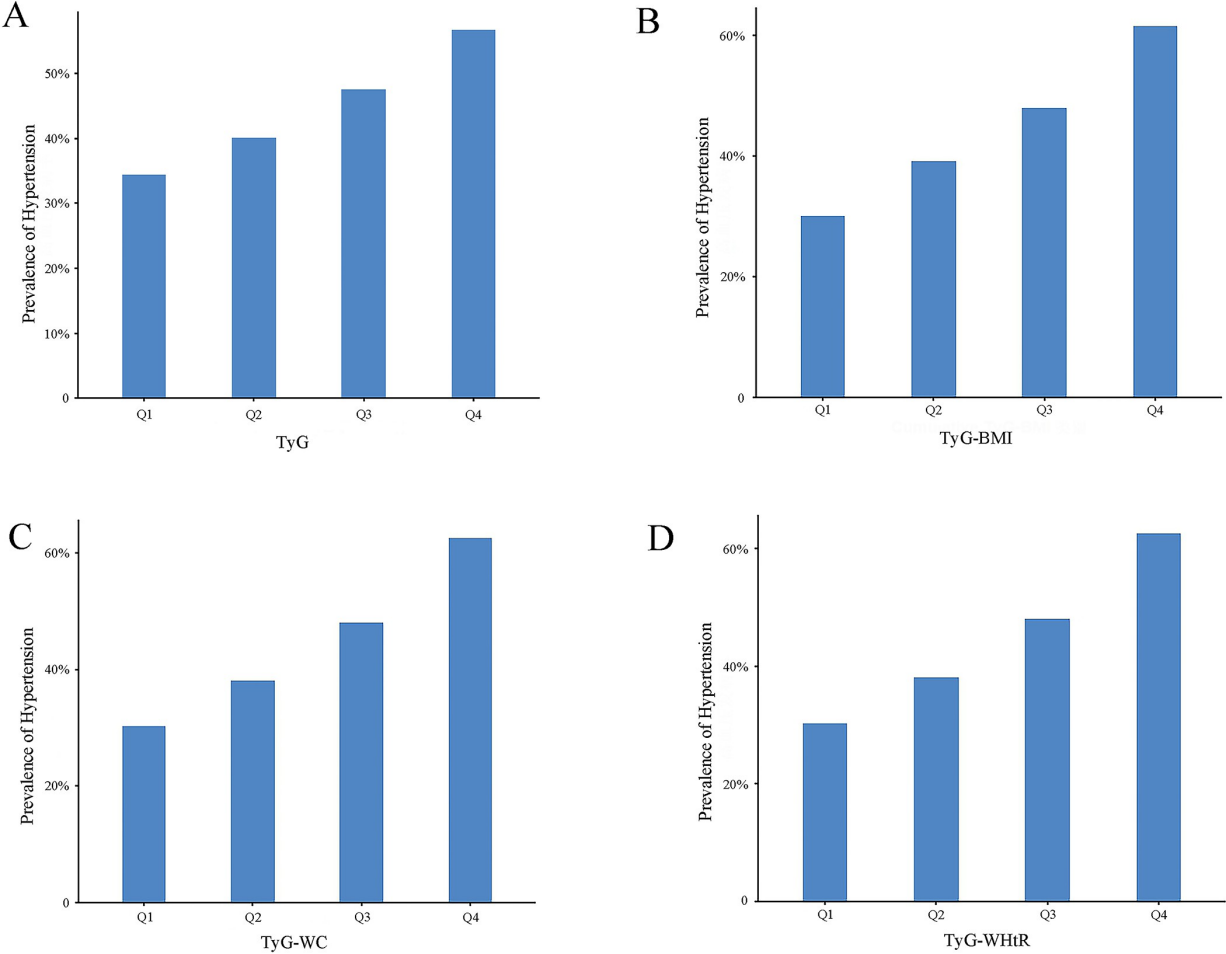
TyG index and its prevalence of hypertension combined with obesity indicators. A) The prevalence of hypertension in TyG quartiles; B) The prevalence of hypertension in TyG-BMI quartiles; C) The prevalence of hypertension in TyG-WC quartiles; D) TyG- Prevalence of hypertension by WHtR quartiles.

This study included a total of 4,135 patients with hypertension, revealing a prevalence rate of 44.71%. The prevalence of hypertension exhibited an increasing trend across the quartiles of TyG, TyG-BMI, TyG-WC, and TyG-WHtR. Specifically, the prevalence of TyG hypertension at the Q1 to Q4 levels was 34.43%, 40.14%, 47.50%, and 56.67%, respectively. For TyG-BMI, the prevalence rates were 30.06%, 39.19%, 48.05%, and 61.55%, respectively. The prevalence rates for TyG-WC were 30.32%, 38.11%, 47.97%, and 62.46%, respectively, while for TyG-WHtR, the rates were 30.23%, 39.36%, 46.54%, and 62.72%, respectively. For further details, please refer to Fig 2.

### Logistic regression analysis of TyG index and its combination of obesity indicators and risk of hypertension

Univariate logistic regression analysis results indicated that, compared to TyG at Q1 level, TyG-BMI, TyG-WC, and TyG-WHtR at Q2-Q4 levels all exhibited an increased risk of hypertension (P < 0.001). After fully adjusting for covariates, TyG at Q2-Q4 levels demonstrated a heightened risk of hypertension relative to Q1 level (Q2: OR = 1.20, 95% CI: 1.05–1.36; Q3: OR = 1.49, 95% CI: 1.30–1.72; Q4: OR = 1.80, 95% CI: 1.48–2.19). Similarly, in comparison to the Q1 level of TyG-BMI, higher levels of TyG-BMI significantly increased the risk of hypertension (Q2: OR = 1.78, 95% CI: 1.56–2.03; Q3: OR = 2.84, 95% CI: 2.46–3.27; Q4: OR = 5.09, 95% CI: 4.33–5.98). When comparing TyG-WC at Q1 level, the Q2-Q4 levels of TyG-WC also showed an increased risk of hypertension (Q2: OR = 1.51, 95% CI: 1.33–1.72; Q3: OR = 2.33, 95% CI: 2.03–2.67; Q4: OR = 3.96, 95% CI: 3.38–4.65). Furthermore, TyG-WHtR at Q2-Q4 levels indicated an increased risk of hypertension compared to Q1 level (Q2: OR = 1.61, 95% CI: 1.41–1.84; Q3: OR = 2.18, 95% CI: 1.90–2.51; Q4: OR = 3.91, 95% CI: 3.33–4.60). For further details, please refer to Table 2.

**Table 2:** Logistic regression analysis of TyG index and its combination with obesity indicators and risk of hypertension.

	Unadjusted	*P*	Model 1^a^	P	Model 2^b^	P
	OR (95%*CI*)		OR (95%CI)		OR (95%*CI*)	
TyG						
Q1	Reference		Reference		Reference	
Q2	1.28 (1.13,1.44)	<0.001	1.29 (1.14,1.46)	<0.001	1.20 (1.05,1.36)	0.006
Q3	1.72 (1.53,1.94)	<0.001	1.81 (1.60,2.05)	<0.001	1.49 (1.30,1.72)	<0.001
Q4	2.49 (2.1,2.81)	<0.001	2.77 (2.45,3.14)	<0.001	1.80 (1.48,2.19)	<0.001
TyG-BMI						
Q1	Reference		Reference		Reference	
Q2	1.50 (1.33,1.69)	<0.001	1.78 (1.57,2.02)	<0.001	1.78 (1.56,2.03)	<0.001
Q3	2.15 (1.91,2.43)	<0.001	2.90 (2.54,3.30)	<0.001	2.84 (2.46,3.27)	<0.001
Q4	3.72 (3.30,4.21)	<0.001	5.59 (4.89,6.40)	<0.001	5.09 (4.33,5.98)	<0.001
TyG-WC						
Q1	Reference		Reference		Reference	
Q2	1.42 (1.25,1.60)	<0.001	1.53 (1.35,1.73)	<0.001	1.51 (1.33,1.72)	<0.001
Q3	2.12 (1.88,2.39)	<0.001	2.41 (2.13,2.74)	<0.001	2.33 (2.03,2.67)	<0.001
Q4	3.82 (3.38,4.32)	<0.001	4.54 (4.00,5.17)	<0.001	3.96 (3.38,4.65)	<0.001
TyG-WHtR						
Q1	Reference		Reference		Reference	
Q2	1.50 (1.33,1.69)	<0.001	1.65 (1.46,1.88)	<0.001	1.61 (1.41,1.84)	<0.001
Q3	2.01 (1.78,2.27)	<0.001	2.35 (2.07,2.67)	<0.001	2.18 (1.90,2.51)	<0.001
Q4	3.88 (3.43,4.39)	<0.001	4.69 (4.11,5.35)	<0.001	3.91 (3.33,4.60)	<0.001

a: Adjust sex, age, marital status, residence, education level.

b: Based on model 1, the smoking status, drinking status, history of dyslipidemia, history of diabetes, LDL-C, HDL-C, TC, UA, and HbA1c were adjusted.

### Dose-response relationship between TyG index and its combined obesity index and risk of hypertension

**Fig 3:**
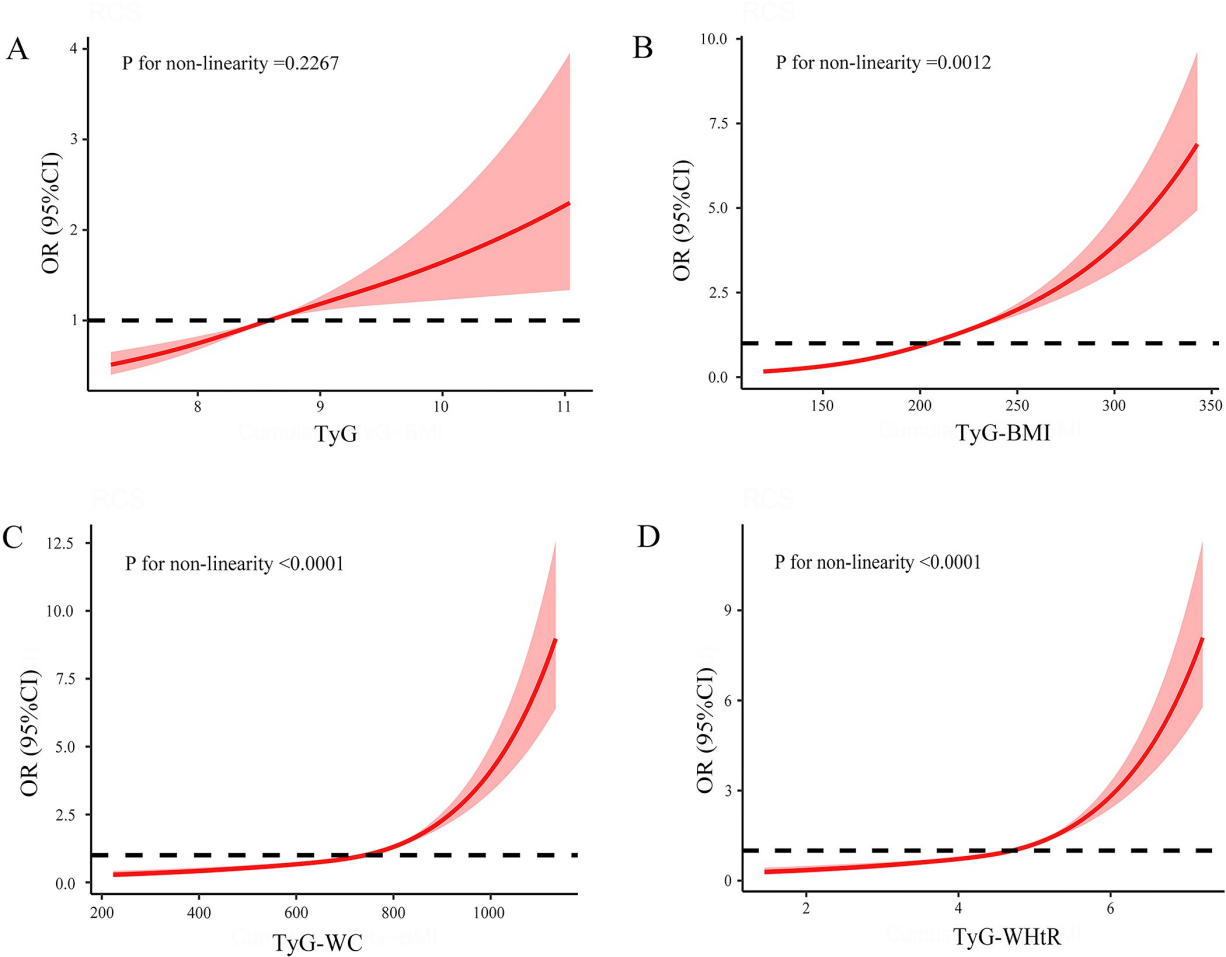
Dose-response relationship between TyG index and its combination with obesity indicators and the risk of hypertension. Adjusted for gender, age, marital status, residential address, education level, smoking status, drinking status, history of dyslipidemia, history of diabetes, LDL-C, HDL-C, TC, UA, and HbA1c: A) the dose-response relationship between TyG and the risk of hypertension; B) the dose-response relationship between TyG-BMI and the risk of hypertension; C) the dose-response relationship between TyG-WC and the risk of hypertension; D) the dose-response relationship between TyG-WHtR and the risk of hypertension.

A restricted cubic spline model was constructed to investigate the dose-response relationship between various TyG indexes, obesity-related indicators, and the risk of hypertension. The results indicated a linear correlation between TyG and the risk of hypertension (P for non-linearity = 0.2267). Additionally, non-linear correlations were observed between TyG-BMI, TyG-WC, and TyG-WHtR with the risk of hypertension(TyG-BMI: P for non-linearity = 0.0012; TyG-WC: P for non-linearity<0.0001; TyG-WHtR: P for non-linearity <0.0001). For further details, please refer to Fig 3.

### ROC curve between TyG index and its combined obesity index and the prevalence of hypertension

**Fig 4:**
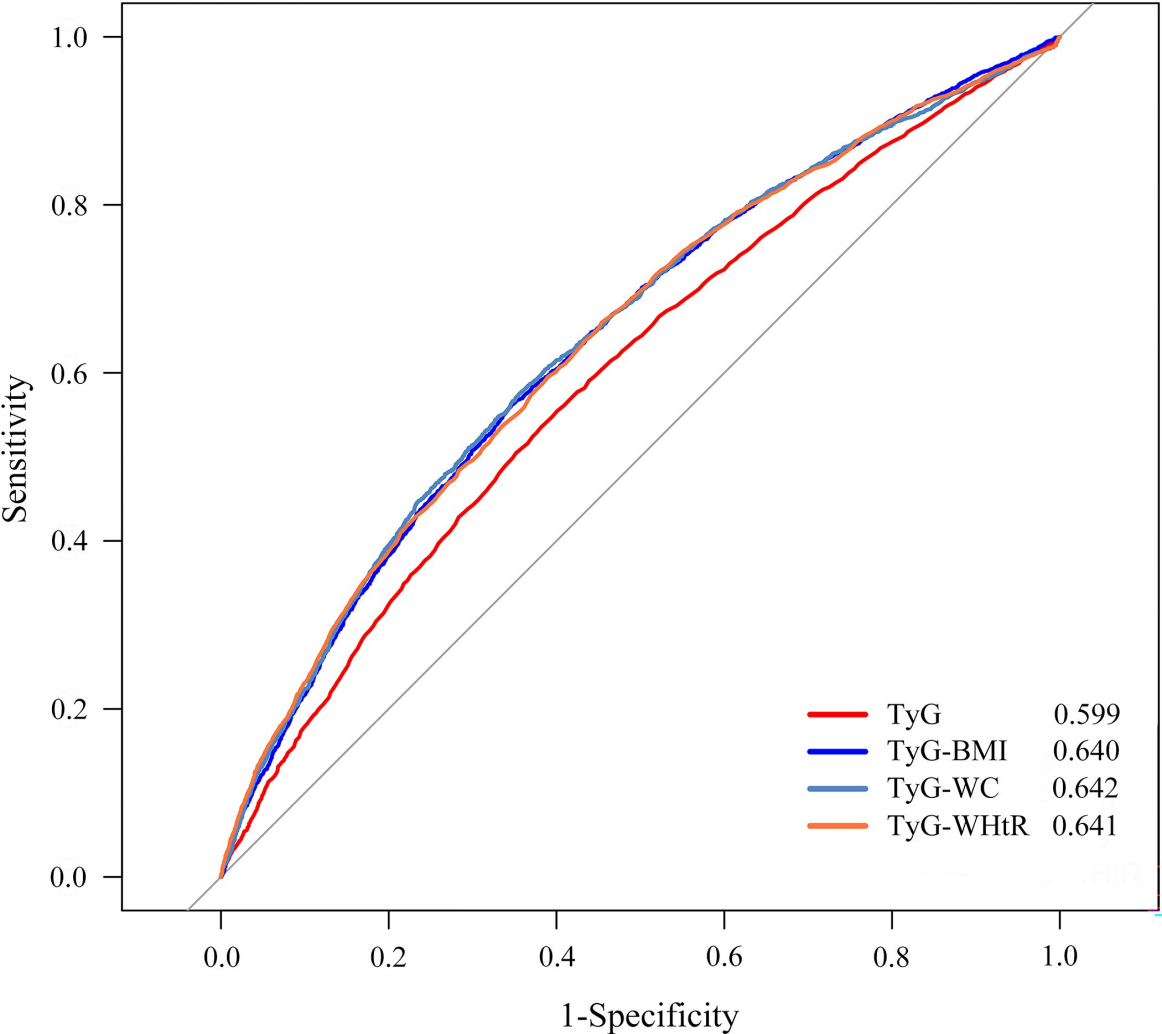
ROC curve between TyG index and its combined obesity index and the prevalence of hypertension.

The ROC curve indicates that TyG-WC exhibits the highest diagnostic performance for hypertension, with an AUC of 0.642 (95% CI: 0.631–0.654). This is followed closely by TyG-WHtR, which has an AUC of 0.641 (95% CI: 0.630–0.652), and TyG-BMI, with an AUC of 0.640 (95% CI: 0.629–0.652). In comparison, TyG demonstrates a lower AUC of 0.599 (95% CI: 0.588–0.611). For further details, please refer to Fig 4.

## Discussion

This study utilizes data from the 2015 CHARLS project, focusing on TyG and obesity as evaluation indicators. These indicators are combined to create TyG-BMI, TyG-WC, and TyG-WHtR. A cross-sectional study design was employed to investigate the relationship between TyG, TyG-BMI, TyG-WC, and TyG-WHtR with the risk of hypertension. The findings indicate that higher levels (Q2-Q4) of TyG, TyG-BMI, TyG-WC, and TyG-WHtR significantly increase the risk of hypertension (P < 0.05). Additionally, TyG demonstrates a linear relationship with the risk of hypertension, whereas TyG-BMI, TyG-WC, and TyG-WHtR exhibit a non-linear relationship. Among these indicators, TyG-WC shows the strongest predictive ability for the prevalence of hypertension.

This study found that insulin resistance plays a significant role in the development of hypertension. As TyG levels gradually increase, the prevalence of hypertension exhibits an upward trend. Elevated TyG levels substantially heighten the risk of hypertension, with odds ratios (OR) for quartiles Q2, Q3, and Q4 reported as 1.20 (95% CI: 1.05–1.36), 1.49 (95% CI: 1.30–1.72), and 1.80 (95% CI: 1.48–2.19), respectively. A 9-year longitudinal study indicated that the cumulative incidence of hypertension over the study period was 43.7%, with the incidence of hypertension associated with the TyG index across quartiles Q1 to Q4 ranging from 28.5% to 59.8%. Cox regression analyses further demonstrated that higher TyG index levels were correlated with an increased risk of hypertension [10]. These findings align with the results of the current study. A cross-sectional study conducted within a Japanese normoglycemic population revealed that the prevalence of hypertension or prehypertension progressively increased with higher TyG quartiles, establishing a positive association between the TyG index and the risk of hypertension or prehypertension [22]. Both studies corroborate the findings of this research. Some scholars propose that hyperinsulinemia resulting from insulin resistance may enhance the activity of the renin-angiotensin-aldosterone system, thereby contributing to elevated blood pressure [23]. Additionally, insulin resistance can stimulate the sympathetic nervous system, promoting the secretion of epinephrine and norepinephrine, which may lead to vascular smooth muscle cell hypertrophy and endothelial dysfunction. This cascade of events can subsequently increase cardiac output and peripheral vascular resistance, ultimately resulting in elevated blood pressure [24].

Previous studies have established that obesity is an independent risk factor for hypertension [25,26]. Body Mass Index (BMI), Waist Circumference (WC), and Waist-to-Height Ratio (WHtR) are simple, cost-effective, and non-invasive anthropometric parameters frequently employed as effective indicators of obesity and associated metabolic risks [27]. In recent years, the integration of TyG with obesity indicators has been increasingly proposed. Research conducted by Maobin Kuang et al. [28] demonstrated that the combination of BMI, WC, and WHtR with TyG can enhance the predictive capacity for insulin resistance. This study examined the impact of various combined indicators of TyG and obesity on the risk of hypertension. The findings revealed that TyG-BMI, TyG-WC, and TyG-WHtR significantly elevated the risk of hypertension (P<0.05). A study utilizing data from the National Health and Nutrition Examination Survey (1999–2018) [29] indicated that the highest quartile levels of TyG-WC and TyG-WHtR were associated with increased all-cause mortality in the US metabolic syndrome population after comprehensive adjustment for covariates (TyG-WC: HR = 1.17, 95% CI: 1.03–1.34; TyG-WHtR: HR = 1.29, 95% CI: 1.13–1.47). This relationship exhibited a nonlinear association, mirroring the results of the present study. Furthermore, Nan Peng et al. [30] found that, compared to the lowest quartile of TyG-BMI, the highest quartile increased the risk of hypertension by 5.27 times (OR = 5.26, 95% CI: 4.46–6.20), which is consistent with the findings of this study.

This study found that TyG-WC exhibits the highest predictive ability for hypertension (AUC: 0.642, 95% CI: 0.631–0.654), followed by TyG-WHtR, TyG-BMI, and TyG. Body Mass Index (BMI) serves as an indicator of overall obesity, while Waist Circumference (WC) and Waist-to-Height Ratio (WtHR) are indicators of central obesity [27]. Obesity is recognized as a significant risk factor for numerous chronic diseases, with central obesity posing a particularly important health challenge, as it is associated with glucose intolerance, insulin resistance, metabolic disorders, and cardiovascular and cerebrovascular diseases [31]. Results from a national cross-sectional study indicated that the rates of general obesity and central obesity among Chinese adult residents were 15.23% and 39.80%, respectively [32]. Simar S. Bajaj et al. highlighted that the Asian population is predominantly affected by central obesity, which increases the risk of type 2 diabetes, cardiovascular disease, and other complications, even at lower BMI levels [33]. Furthermore, Zhang et al. [34] found that among TyG, TyG-WC, TyG-BMI, and TyG-WHtR, TyG-WC had the highest predictive accuracy for prediabetes and diabetes, corroborating the findings of this study. The levels of fasting free fatty acids (FFA) in the plasma of individuals with central obesity are elevated, promoting the fatification of non-adipose tissue, which produces lipotoxic effects on pancreatic beta cells, leading to inhibited insulin secretion and, consequently, inducing insulin resistance [35]. This resistance further contributes to elevated blood pressure. Additionally, Cheng et al.’s study of 7,217 metabolically normal obese individuals, utilizing data from the US National Health and Nutrition Examination Survey from 2001 to 2014, revealed that for every 10 cm increase in WC, the risk of hypertension in this population rose by 24% [36]. Therefore, TyG-WC may serve as an effective indicator for predicting the prevalence of hypertension among the elderly.

This study has several limitations. (1) As a cross-sectional study, it cannot establish causal associations between the various TyG indexes, obesity-related indicators, and the prevalence of hypertension. (2) Although we have accounted for general information, behavioral habits, disease history, and other factors, there may still be unexamined confounding variables that could influence the research outcomes. (3) The study population consisted of middle-aged and elderly individuals aged 45 and above, which limits the applicability of the predictive ability of TyG-WC for hypertension to younger demographics.

In summary, there are varying degrees of correlation between TyG, TyG-WC, TyG-BMI, and TyG-WHtR and the risk of hypertension among middle-aged and elderly individuals in my country. As the levels of these indicators increase, the risk of hypertension also rises. Notably, TyG-WC demonstrates the strongest diagnostic capability for hypertension. Therefore, enhancing the management of TyG-WC in middle-aged and elderly populations in my country could effectively delay the onset of hypertension.

## Supporting information

S1 Appendix Tables S1-S3. (DOCX)

## Abbreviations

TyG: Triglyceride-Glucose Index
TyG-BMI: Triglyceride glucose -body mass index
TyG-WC: Triglyceride glucose -waist circumference
TyG-WHtR: Triglyceride glucose -waist height ratio
TG: Triglyceride
HDL-C: High-density lipoprotein cholesterol
LDL-C: Low-density lipoprotein cholesterol
FPG: Fasting plasma glucose
ROC: Receiver operating characteristic
AUC: Area under curve
TC: Total cholesterol
HbA1c: Glycosylated hemoglobin
UA: Uric acid
BMI: Body mass index
WHtR: Waist height ratio
WC: waist circumference
OR: Odds ratios
CI: Confidence intervals
