# Supplementary material for: Association between the triglyceride-glucose index and its combined obesity indicators and the risk of hypertension in middle-aged and older Chinese adults: A nationwide cross-sectional study
Source: PLoS One. 2025 Jan 2;20(1):e0316581. doi: 10.1371/journal.pone.0316581 (PMC11694962; doi:10.1371/journal.pone.0316581)
Supplement: S1 Appendix — (DOCX) [file pone.0316581.s001.docx]

Table S1 Basic characteristics of different TyG-BMI quartiles

| **Variable** | **Overall** | **TyG-BMI quartiles** | | | | **P** |
| --- | --- | --- | --- | --- | --- | --- |
|  |  | **Q1** | **Q2** | **Q3** | **Q4** |  |
| Sex |  |  |  |  |  | <0.001 |
| Men | 4,274 (46.21) | 1,310 (56.66) | 1,133 (49.01) | 944 (40.83) | 887 (38.37) |  |
| Women | 4,974 (53.79) | 1,002(43.34) | 1,179 (50.99) | 1,368 (59.17) | 1,425 (61.63) |  |
| Residence |  |  |  |  |  | <0.001 |
| City | 1,886 (20.39) | 317 (13.71) | 415 (17.95) | 560 (24.22) | 594 (25.69) |  |
| Rural | 7,362 (79.61) | 1,995 (86.29) | 1,897 (82.05) | 1,752 (75.78) | 1,718 (74.31) |  |
| Education |  |  |  |  |  | <0.001 |
| Illiteracy | 2,382 (25.75) | 674 (29.15) | 593 (25.65) | 567 (24.52) | 548 (23.70) |  |
| Primary school | 3,904 (42.21) | 1,031 (44.59) | 980 (42.39) | 952 (41.18) | 941 (40.70) |  |
| Middle school  and above | 2,962 (32.03) | 607 (26.25) | 739 (31.96) | 793 (34.30) | 823 (35.60) |  |
| Marital status |  |  |  |  |  | <0.001 |
| Married | 7,969 (86.17) | 1,932 (83.56) | 1,943 (84.04) | 2,018 (87.28) | 2,076 (89.79) |  |
| Others | 1,279 (13.82) | 380 (16.44) | 369 (15.96) | 294 (12.72) | 236 (10.21) |  |
| Smoking status |  |  |  |  |  | <0.001 |
| No | 5,503 (59.50) | 1,099 (47.53) | 1,338 (57.87) | 1,517 (65.61) | 1,549 (67.00) |  |
| Yes | 2,736 (29.58) | 971 (42.00) | 728 (31.49) | 550 (23.79) | 487 (21.06) |  |
| Quit smoking | 1,009 (10.92) | 242 (10.47) | 246 (10.64) | 245 (10.60) | 276 (11.94) |  |
| Drinking status |  |  |  |  |  | <0.001 |
| No | 6,039 (65.30) | 1,400 (60.55) | 1,454 (62.89) | 1,569 (67.86) | 1,616 (69.90) |  |
| Yes | 3,209 (34.70) | 912 (39.45) | 858 (37.11) | 743 (32.14) | 696 (30.10) |  |
| Hypertension |  |  |  |  |  | <0.001 |
| No | 5,113 (55.29) | 1,617 (69.94) | 1,406 (60.81) | 1,201 (51.95) | 889 (38.45) |  |
| Yes | 4,135 (44.71) | 695 (30.06) | 906 (39.19) | 1,111(48.05) | 1,423 (61.55) |  |
| Dyslipidemia |  |  |  |  |  | <0.001 |
| No | 5,549 (60.00) | 1,977 (85.51) | 1,679 (72.62) | 1,244 (53.81) | 649 (28.07) |  |
| Yes | 3,699 (40.00) | 335 (14.49) | 633 (27.38) | 1,068 (46.19) | 1,663 (71.93) |  |
| Diabetes |  |  |  |  |  | <0.001 |
| No | 8,008 (86.59) | 2,191 (94.77) | 2,118 (91.61) | 1,977 (85.51) | 1,722 (74.48) |  |
| Yes | 1,240 (13.41) | 121 (5.23) | 194 (8.39) | 335 (14.49) | 590 (25.52) |  |
| Age | 61.38$\pm$9.28 | 63.63$\pm$9.92 | 61.74$\pm$9.31 | 60.61$\pm$8.90 | 59.53$\pm$8.44 | <0.001 |
| BMI | 23.83$\pm$3.54 | 19.66$\pm$1.59 | 22.66$\pm$1.27 | 24.88$\pm$1.47 | 28.10$\pm$2.39 | <0.001 |
| WC | 85.76$\pm$11.11 | 75.53$\pm$6.68 | 83.19$\pm$7.11 | 88.40$\pm$9.40 | 95.92$\pm$9.43 | <0.001 |
| WHtR | 0.54$\pm$0.07 | 0.48$\pm$0.04 | 0.52$\pm$0.05 | 0.56$\pm$0.06 | 0.61$\pm$0.06 | <0.001 |
| HDL-C | 51.44$\pm$11.53 | 56.72$\pm$12.76 | 53.07$\pm$11.50 | 49.44$\pm$9.71 | 46.55$\pm$9.19 | <0.001 |
| LDL-C | 102.94$\pm28.94$ | 96.17$\pm$28.07 | 104.07$\pm$27.58 | 106.54$\pm$29.15 | 104.97$\pm$29.79 | <0.001 |
| TC | 184.52$\pm$36.50 | 173.94$\pm$34.58 | 182.02$\pm$33.69 | 187.15$\pm$36.86 | 194.99$\pm$37.55 | <0.001 |
| FPG | 100.56$\pm$30.11 | 91.12$\pm$18.31 | 96.07$\pm$22.42 | 101.07$\pm$27.00 | 113.97$\pm$30.11 | <0.001 |
| UA | 4.92$\pm$1.41 | 4.63$\pm$1.33 | 4.79$\pm$1.38 | 4.95$\pm$1.36 | 5.33$\pm$1.45 | <0.001 |
| HbA1c | 5.99$\pm$1.00 | 5.76$\pm$0.71 | 5.83$\pm$0.75 | 6.00$\pm$0.92 | 6.38$\pm$1.37 | <0.001 |
| TG | 111.50  [81.42,163.72] | 77.88  [62.83,98.23] | 97.35  [78.76,125.66] | 125.66  [96.46,168.14] | 179.65  [132.74,263.72] | <0.001 |

Table S2 Basic characteristics of different TyG-WC quartiles

| **Variable** | **Overall** | **TyG-WC quartiles** | | | | **P** |
| --- | --- | --- | --- | --- | --- | --- |
|  |  | **Q1** | **Q2** | **Q3** | **Q4** |  |
| Sex |  |  |  |  |  | <0.001 |
| Men | 4,274 (46.21) | 1,216 (52.60) | 1,077 (46.58) | 971 (42.00) | 1,010 (43.69) |  |
| Women | 4,974 (53.79) | 1,096 (47.40) | 1,235 (53.42) | 1,341 (58.00) | 1,302 (56.31) |  |
| residence |  |  |  |  |  | <0.001 |
| City | 1,886 (20.39) | 324 (14.01) | 405 (17.52) | 534 (23.10) | 623 (26.95) |  |
| Rural | 7,362 (79.61) | 1,988 (85.99) | 1,907 (82.48) | 1,778 (76.90) | 1,689 (73.05) |  |
| Education |  |  |  |  |  | <0.001 |
| Illiteracy | 2,382 (25.75) | 648 (28.03) | 635 (27.47) | 536 (23.18) | 563 (24.35) |  |
| Primary school | 3,904 (42.21) | 1,024 (44.29) | 981 (42.43) | 991 (42.86) | 908 (39.27) |  |
| Middle school  and above | 2,962 (32.03) | 640 (27.68) | 696 (30.10) | 785 (33.95) | 841 (36.38) |  |
| Marital status |  |  |  |  |  | <0.001 |
| Married | 7,969 (86.17) | 1,961 (84.82) | 1,971 (85.25) | 1,993 (86.20) | 2,044 (88.41) |  |
| Others | 1,279 (13.82) | 351 (15.18) | 341 (14.75) | 319 (13.80) | 268 (11.59) |  |
| Smoking status |  |  |  |  |  | <0.001 |
| No | 5,503 (59.50) | 1,210 (52.34) | 1,373 (59.69) | 1,475 (63.80) | 1,445 (62.50) |  |
| Yes | 2,736 (29.58) | 891 (38.54) | 701 (30.32) | 582 (25.17) | 562 (24.31) |  |
| Quit smoking | 1,009 (10.92) | 211 (9.13) | 238 (10.29) | 255 (11.03) | 305 (13.19) |  |
| Drinking status |  |  |  |  |  | <0.001 |
| No | 6,039 (65.30) | 1,448 (62.63) | 1,467 (63.45) | 1,588 (68.69) | 1,536 (66.44) |  |
| Yes | 3,209 (34.70) | 864 (37.37) | 845 (36.55) | 724 (31.31) | 776 (33.56) |  |
| Hypertension |  |  |  |  |  | <0.001 |
| No | 5,113 (55.29) | 1,611 (69.68) | 1,431 (61.89) | 1,203 (52.03) | 868 (37.54) |  |
| Yes | 4,135 (44.71) | 701 (30.32) | 881 (38.11) | 1,109 (47.97) | 1,444 (62.46) |  |
| Dyslipidemia |  |  |  |  |  | <0.001 |
| No | 5,549 (60.00) | 2,000 (86.51) | 1,717 (74.26) | 1,261 (54.54) | 571 (24.70) |  |
| Yes | 3,699 (40.00) | 312 (13.49) | 595 (25.74) | 1,051 (45.46) | 1,741 (75.30) |  |
| Diabetes |  |  |  |  |  | <0.001 |
| No | 8,008 (86.59) | 2,201 (95.20) | 2,143 (92.69) | 2,005 (86.72) | 1,659 (71.76) |  |
| Yes | 1,240 (13.41) | 111 (4.80) | 169 (7.31) | 307 (13.28) | 653 (28.24) |  |
| Age | 61.38$\pm$9.28 | 63.63$\pm$9.92 | 61.74$\pm$9.31 | 60.61$\pm$8.89 | 59.53$\pm$8.44 | <0.001 |
| BMI | 23.83$\pm$3.54 | 19.66$\pm$1.59 | 22.66$\pm$1.27 | 24.88$\pm$1.47 | 28.09$\pm$2.39 | <0.001 |
| WC | 85.76$\pm$11.11 | 75.53$\pm$6.68 | 83.19$\pm$7.11 | 88.40$\pm$9.40 | 95.92$\pm$9.43 | <0.001 |
| WHtR | 0.54$\pm$0.07 | 0.48$\pm$0.04 | 0.53$\pm$0.05 | 0.56$\pm$0.06 | 0.61$\pm$0.06 | <0.001 |
| HDL-C | 51.44$\pm$11.53 | 56.72$\pm$12.76 | 53.07$\pm$11.50 | 49.44$\pm$9.71 | 46.54$\pm$9.19 | <0.001 |
| LDL-C | 102.94$\pm$28.94 | 96.17$\pm$28.07 | 104.07$\pm$27.58 | 106.53$\pm$29.15 | 104.97$\pm$29.79 | <0.001 |
| TC | 184.52$\pm$36.50 | 173.94$\pm$34.55 | 182.02$\pm$33.69 | 187.14$\pm$36.86 | 194.99$\pm$37.55 | <0.001 |
| FPG | 100.56$\pm$30.11 | 91.12$\pm$18.31 | 96.08$\pm$22.42 | 101.07$\pm$27.00 | 113.97$\pm$42.09 | <0.001 |
| UA | 4.92$\pm$1.41 | 4.63$\pm$1.33 | 4.79$\pm$1.38 | 4.95$\pm$1.36 | 5.33$\pm$1.45 | <0.001 |
| HbA1c | 5.99$\pm$1.00 | 5.76$\pm$0.71 | 5.83$\pm$0.75 | 6.00$\pm$0.92 | 6.38$\pm$1.37 | <0.001 |
| TG | 111.50  [81.42,163.72] | 76.11  [61.95,96.46] | 96.46  [77.88,122.12] | 126.55  [97.35,165.49] | 185.84  [138.94,270.58] | <0.001 |

Table S3 Basic characteristics of different TyG-WHtR quartiles

| **Variable** | **Overall** | **TyG-****WHtR quartiles** | | | | **P** |
| --- | --- | --- | --- | --- | --- | --- |
|  |  | **Q1** | **Q2** | **Q3** | **Q4** |  |
| Sex |  |  |  |  |  | <0.001 |
| Men | 4,274 (46.21) | 1,513 (65.44) | 1,167 (50.48) | 908 (39.27) | 686 (29.67) |  |
| Women | 4,974 (53.79) | 799 (34.56) | 1,145 (49.52) | 1,404 (60.73) | 1,626 (70.33) |  |
| residence |  |  |  |  |  | <0.001 |
| City | 1,886 (20.39) | 341 (14.75) | 428 (18.51) | 552 (23.88) | 565 (24.44) |  |
| Rural | 7,362 (79.61) | 1,971 (85.25) | 1,884 (81.49) | 1,760 (76.12) | 1,747 (75.56) |  |
| Education |  |  |  |  |  | <0.001 |
| Illiteracy | 2,382 (25.75) | 543 (23.49) | 568 (24.57) | 566 (24.48) | 705 (30.49) |  |
| Primary school | 3,904 (42.21) | 1,035 (44.77) | 987 (42.69) | 958 (41.44) | 924 (39.97) |  |
| Middle school  and above | 2,962 (32.03) | 734 (31.75) | 757 (32.74) | 788 (34.08) | 683 (29.54) |  |
| Marital status |  |  |  |  |  | <0.001 |
| Married | 7,969 (86.17) | 1,999 (86.46) | 1,999 (86.46) | 1,980 (85.64) | 1,991 (86.12) |  |
| Others | 1,279 (13.82) | 313 (13.54) | 313 (13.54) | 332 (14.36) | 321 (13.88) |  |
| Smoking status |  |  |  |  |  | <0.001 |
| No | 5,503 (59.50) | 1,005 (43.47) | 1,297 (56.10) | 1,532 (66.26) | 1,669 (72.19) |  |
| Yes | 2,736 (29.58) | 1,052 (45.50) | 737 (31.88) | 529 (22.88) | 418 (18.08) |  |
| Quit smoking | 1,009 (10.92) | 255 (11.03) | 278 (12.02) | 251 (10.86) | 225 (9.73) |  |
| Drinking status |  |  |  |  |  | <0.001 |
| No | 6,039 (65.30) | 1,303 (56.36) | 1,452 (62.80) | 1,569 (67.86) | 1,715 (74.18) |  |
| Yes | 3,209 (34.70) | 1,009 (43.64) | 860 (37.20) | 743 (32.14) | 597 (25.82) |  |
| Hypertension |  |  |  |  |  | <0.001 |
| No | 5,113 (55.29) | 1,613 (69.77) | 1,402 (60.64) | 1,236 (53.46) | 862 (37.28) |  |
| Yes | 4,135 (44.71) | 699 (30.23) | 910 (39.36) | 1,076 (46.54) | 1,450 (62.72) |  |
| Dyslipidemia |  |  |  |  |  | <0.001 |
| No | 5,549 (60.00) | 2,000 (86.51) | 1,701 (73.57) | 1,232 (53.29) | 616 (26.64) |  |
| Yes | 3,699 (40.00) | 312 (13.49) | 611 (26.43) | 1,080 (46.71) | 1,696 (73.36) |  |
| Diabetes |  |  |  |  |  | <0.001 |
| No | 8,008 (86.59) | 2,197 (95.03) | 2,136 (92.39) | 2,006 (86.76) | 1,669 (72.19) |  |
| Yes | 1,240 (13.41) | 115 (4.97) | 176 (7.61) | 306 (13.24) | 643 (27.81) |  |
| Age | 61.38$\pm$9.28 | 61.70$\pm$9.59 | 61.12$\pm$9.25 | 61.01$\pm$9.16 | 61.68$\pm$9.10 | <0.001 |
| BMI | 23.83$\pm$3.54 | 20.47$\pm$2.39 | 22.79$\pm$2.22 | 24.78$\pm$2.41 | 27.27$\pm$2.94 | <0.001 |
| WC | 85.76$\pm$11.11 | 73.53$\pm$9.84 | 83.39$\pm$5.49 | 89.44$\pm$5.93 | 96.67$\pm$6.62 | <0.001 |
| WHtR | 0.54$\pm$0.07 | 0.46$\pm$0.06 | 0.53$\pm$0.03 | 0.57$\pm$0.03 | 0.62$\pm$0.04 | <0.001 |
| HDL-C | 51.44$\pm$11.53 | 56.49$\pm$12.74 | 52.65$\pm$11.44 | 49.69$\pm$10.06 | 46.93$\pm$9.34 | <0.001 |
| LDL-C | 102.94$\pm$28.94 | 95.15$\pm$27.01 | 104.15$\pm$27.60 | 107.29$\pm$29.01 | 105.15$\pm$30.52 | <0.001 |
| TC | 184.52$\pm$36.50 | 172.27$\pm$33.22 | 181.31$\pm$33.94 | 187.75$\pm$35.00 | 196.77$\pm$39.12 | <0.001 |
| FPG | 100.56$\pm$30.11 | 90.95$\pm$17.56 | 95.31$\pm$20.20 | 99.99$\pm$24.27 | 115.99$\pm$44.32 | <0.001 |
| UA | 4.92$\pm$1.41 | 4.69$\pm$1.33 | 4.82$\pm$1.41 | 4.94$\pm$1.39 | 5.25$\pm$1.43 | <0.001 |
| HbA1c | 5.99$\pm$1.00 | 5.72$\pm$0.63 | 5.82$\pm$0.70 | 5.97$\pm$0.89 | 6.46$\pm$1.42 | <0.001 |
| TG | 111.50  [81.42, 163.72] | 76.11  [61.95,95.58] | 96.46  [78.76,124.77] | 125.66  [96.46,169.03] | 184.95  [138.05,269.91] | <0.001 |
